# Effects of Vaccination Against Influenza, Pertussis, and COVID-19 on Human Milk Antibodies: Current Evidence and Implications for Health Equity

**DOI:** 10.3389/fimmu.2022.910383

**Published:** 2022-07-12

**Authors:** Soumya Hunagund, Yarden Golan, Ifeyinwa V. Asiodu, Mary Prahl, Stephanie L. Gaw

**Affiliations:** ^1^ Division of Maternal-Fetal Medicine, Department of Obstetrics, Gynecology, and Reproductive Sciences, University of California, San Francisco, San Francisco, CA, United States; ^2^ Department of Bioengineering and Therapeutic Sciences, University of California, San Francisco, San Francisco, CA, United States; ^3^ Institute for Human Genetics, University of California, San Francisco, San Francisco, CA, United States; ^4^ Department of Family Health Care Nursing, University of California, San Francisco, San Francisco, CA, United States; ^5^ Department of Pediatrics, University of California, San Francisco, San Francisco, CA, United States; ^6^ Division of Pediatric Infectious Diseases and Global Health, University of California, San Francisco, San Francisco, CA, United States; ^7^ Center for Reproductive Sciences, Department of Obstetrics, Gynecology, and Reproductive Sciences, University of California, San Francisco, San Francisco, CA, United States

**Keywords:** human milk, COVID - 19, influenza, pertussis, vaccination, infant health, immunization

## Abstract

Human milk contains three antibody classes that confer mucosal immunity to the breastfed infant: secretory IgA (SIgA), secretory IgM (SIgM), and IgG. Influenza and pertussis vaccines administered during pregnancy induce pathogen specific SIgA and IgG responses in human milk that have been shown to protect the breastfed infant from these respiratory illnesses. In addition, mRNA vaccines against the SARS-CoV-2 virus administered during pregnancy and lactation induce anti-SARS-CoV-2 IgG and IgA responses in human milk. This review summarizes the immunologic benefits of influenza, pertussis, and COVID-19 vaccines conferred by human milk. Additionally, future research direction in human milk immunity and public health needs to improve lactational support are discussed.

## Introduction

Human milk has been shown to have numerous benefits for infants (1–5) as well as for breastfeeding mothers which experience short-term and long-term health benefits (5–8). The World Health Organization recommends exclusive breastfeeding for the first six months after birth, and up to two years with the introduction of complementary foods (9). Unfortunately, due to systemic and structural barriers such as racism, lack of workplace accommodations, and inequitable access to human milk feeding resources, breastfeeding disparities and inequities remain (10–12). In general, breastfeeding initiation and duration rates are higher among Asian and White mothers and lower among Black and Indigenous mothers in the U.S (13).

Vaccination during pregnancy and lactation not only has immune protection for the mother, but also provides immunologic benefits for their child through the transfer of immune factors *in utero* and through human milk. Pregnant women and those who have recently given birth may face increased vulnerability to infections and severe illness (14, 15). Thus, vaccines serve as a critical component of preventative healthcare for pregnant and lactating women and an important public health intervention (16, 17). However, inequities and disparities also extend to vaccinations. Presently, in the U.S., children, adolescents, and adults who are uninsured, living in rural communities, have lower levels of income, and identify as a person of color, experience lower rates of recommended vaccination (18–20). Given the benefits and significance of human milk, lactation, and vaccines across the life course, the barriers need to be addressed to make certain that all mothers and infants, especially those most marginalized, have access to critical resources and supports during the perinatal period.

In this review, we discuss the barriers that need to be addressed to improve equity, and summarize the literature regarding humoral immunity in the human milk after influenza and pertussis vaccinations, as well as the latest data on human milk immunity conferred by the mRNA-based COVID-19 vaccines.

## Antibodies in Human Milk

For the first few months of life, the infant’s immune system is immature and they therefore rely on maternal passive immunity for protection and to distinguish pathogenic from commensal bacteria (21). During pregnancy, specific maternal IgG antibodies are transferred from the mother through the placenta to the fetal bloodstream to provide systemic immunity that confers protection for the first few months of infancy. Maternally-derived antibodies gradually decrease during the first year of life while the infant builds protective immune responses through vaccination and early life pathogen exposure (22). After birth, lactating mothers continue to transfer milk-derived antibodies to their newborn which provide passive mucosal immunity. Human milk contains protective immunologic components including immune cells, cytokines, glycoproteins (e.g. lactoferrin), human milk oligosaccharides, and antibodies such as maternal secretory IgA (SIgA), secretory IgM (SIgM), and IgG (21, 23, 24). In humans, mucosal barriers close shortly after birth, and therefore human milk antibodies are prevented from passing into the bloodstream due to decreasing permeability of the gut. As a result, milk antibodies predominately provide mucosal immunity (25, 26).

Serum IgA is a monomer, whereas mucosal IgA is a dimer. The IgA dimers in the mammary gland bind to polymeric immunoglobulin receptor (pIgR) on the basolateral surface of the epithelial cells and travel across the cell to the apical surface (27). There, the external domain of the pIgR bound to the dimeric mucosal IgA is cleaved, and the remaining compound is secreted into the human milk as SIgA (26). SIgA provides first line protection along mucosal surfaces including the respiratory and digestive tracts (27). It has also been shown to be protective against various diarrheal diseases as infants consuming human milk with higher SIgA levels were more likely to be asymptomatic for these diseases (24, 28–31). Pentameric secretory IgM usually produces the primary antibody response to an antigen and activates the complement cascade upon antigen binding. SIgM is delivered to human milk through the same mechanism as SIgA. IgG is the least prominent antibody in human milk. Monomeric IgG from maternal blood is delivered in human milk through binding of the neonatal Fc receptor (FcRn) on epithelial cells in the mammary gland (32–35). Human milk-derived maternal IgG binds intraluminal pathogens in the infant’s gut and helps protect against enteric infections (36, 37). Milk antibodies main functions are summarized in **Table 1**.

**Table 1:** Function and location of human milk antibodies.

Antibodies	Structure	Location	Function
**SIgA**	Dimer	Mucosal sites including respiratory and digestive tracts	-Intracellular neutralization (forming complexes with the viral proteins) -Virus excretion through transcytosis of immune complexes to the intestine lumen. -Immune exclusion to prevent pathogens penetration.
**SIgM**	Pentamer	Mucosal sites including respiratory and digestive tracts	-Intracellular neutralization -Ability to activate complement
**IgG**	Monomer	Primarily in blood	-Pathogenic neutralization including viruses and bacteria

## Antibody Composition in Human milk Post-Influenza and Pertussis Vaccination

### Influenza Vaccination

Influenza (flu) viruses are RNA viruses (38), that can cause severe illness, particularly in pregnant people who are at high risk for infectious complications leading to hospitalization (39). Influenza vaccines are updated annually to optimize protection against circulating influenza viruses that are predicted to be the most common in the upcoming year (40). Currently in the U.S., inactivated virus quadrivalent vaccines are recommended for pregnant individuals, which protect against four different types of flu viruses. There is year-to-year variability in vaccine efficacy, due to a number of factors including antigenic mismatch, pre-existing immunity, and the limited ability to predict the dominant viruses each year. However, the efficiency of the flu vaccines typically ranges between 50-70% in pregnancy but may be less for other populations (41, 42).

Influenza vaccination during pregnancy leads to a 40% decreased risk of influenza-related hospitalization in pregnant women (43), as well as a significant increase in maternal and infant serum influenza IgG levels (44, 45). In addition, numerous studies have shown a decrease in the incidence of influenza in infants born to vaccinated mothers up to 6 months post-delivery (45–48). This protection is mostly attributed to transplacentally-derived IgG antibodies which are transferred during pregnancy, and wanes in the infants typically three to six months after delivery (44, 45, 49–51). However, in breastfed infants, human milk-derived antibodies may also provide additional layer of influenza protection in the infant during the breastfeeding period. A recent study evaluated longitudinal levels of anti-influenza IgA in human milk, samples were collected from lactating individuals after administering the trivalent inactivated influenza vaccine or a 23-valent pneumococcal polysaccharide vaccine (control) to pregnant women in the third trimester (52). Human milk anti-influenza IgA levels in milk were maintained at a significantly higher level in those who received the influenza vaccine for at least 6 months after delivery compared to controls (52). In addition to IgA, anti-influenza IgM and IgG are also present in milk but at lower levels (53). Human milk also contains varying levels of immune cells including innate cells, memory T cells, and plasma B cells (54–58), but limited data exists on the antigen-specificity of these cells and response to infection or vaccination. A prior study demonstrated influenza-specific CD8 T cells in human milk (57, 59). However, the degree of protection conferred by milk immune cells remains unknown. Breastfeeding exclusivity is associated with lower rate of infant febrile respiratory illness (52, 60) compared to non-exclusively breastfed infant. Additional studies are needed to understand the various factors in milk that confer this protection to infants.

### Pertussis Vaccination

Pertussis (also known as whooping cough) is a childhood respiratory illness caused by the bacterium *Bortadella pertussis*. Pertussis (PT) booster immunization during the late second or third trimester of pregnancy is an important public health strategy to reduce the morbidity and mortality from whooping cough in neonates. Since 2010, the pertussis vaccine has been recommended for all pregnant people between 27- and 36-weeks of gestation in order to provide protective antibodies to the fetus for protection against pertussis, in the critical early months of the infant’s life when they are most at risk for serious disease (61, 62). In the U.S., this is typically administered through the combined Tdap vaccine, which also provides protection against tetanus, diphtheria, in addition to pertussis (63).

Studies have demonstrated that after maternal vaccination high levels of anti-PT IgG is present in newborn blood due to transplacental transfer from mother to infant (64). After delivery, pertussis-specific IgA as well as IgG are present in colostrum and mature human milk and are detected for at least 8 weeks postpartum after maternal vaccination during pregnancy (65, 66).

The effectiveness of maternal vaccination in infant protection against PT infection at the first months of life ranges from 88 to 93% (67–70). Further, infants with pertussis whose mothers received the TdaP vaccine had lower risks of hospitalization, ICU admission, and shorter hospital stays compared to mothers who were not vaccinated (71). In summary, vaccination with Tdap during or shortly after pregnancy greatly increases the level of anti-PT antibodies in human milk (64–66, 72, 73) and may contribute to the protection provided to the infant against pertussis infection.

## Immune Responses in Human Milk Following COVID-19 Vaccination

BNT162b2 (BioNTech and Pfizer) and mRNA-1273 (Moderna) are mRNA-based vaccines approved by the Food and Drug Administration (FDA) to use against COVID-19 (74, 75). In addition, two vector-based vaccines AZD1222 (Oxford/AstraZeneca) and Ad26.COV2.S (Johnson & Johnson/Janssen) are widely used worldwide (76–80). However, due to the timing of vaccine approval, there is currently limited data on vector-based vaccines in pregnancy and lactation, and for purposes of this review we will focus on mRNA vaccines. BNT162b2 and mRNA-1273 vaccines contain the mRNA sequence of the SARS-CoV-2 Spike protein, coated by a lipid-nanoparticle envelope. Upon administration, the lipid nanoparticles are absorbed by cells, and the mRNA sequence is released into the cytoplasm, where it is translated into Spike protein that is presented on the cell surface of vaccinated cells. This Spike protein is recognized by immune cells to generate a robust and specific immune response against the Spike protein (81, 82). These vaccines have been found to be highly efficient in prevention of severe COVID-19 disease (83, 84) and to be safe for administration during pregnancy and lactation (85–95).

For mothers that were vaccinated while pregnant, their infants had detectable levels of anti-SARS-CoV-2 IgG antibodies in cord blood and in infant follow up blood samples, demonstrating transfer of these IgG antibodies *via* the placenta to the fetal bloodstream (96–98). Similar to influenza and pertussis vaccination during pregnancy (44, 46), SARS-CoV-2 vaccination during pregnancy reduced the risk of infant hospitalization for COVID-19 up to 4-6 months of age by 30-70% (99, 100). In contrast, infants born to mothers vaccinated after pregnancy did not have anti-SARS-CoV-2 IgG in their blood (25, 101). However, COVID-19 vaccination during pregnancy and lactation both elicited transfer of anti-SARS-CoV-2 antibodies to human milk (25, 96, 97, 102–105).

Since SARS-CoV-2 is a novel pathogen, the implementation of COVID-19 vaccines has provided a unique opportunity to understand primary immune responses in human milk to a novel antigen in lactating people. We have summarized multiple studies that have evaluated mRNA vaccination during lactation and human milk antibodies (**Table 2**). Most studies have found an initial increase of milk IgG 14-21 days after the first dose of vaccine, with further robust increased levels peaking at 7 days after the second dose and remaining elevated for at least 6 weeks (78, 96, 102–105). In most lactating people, 4-10 weeks after the second dose, anti-SARS-CoV-2 IgG levels in milk were still significantly higher compared to their levels before vaccination (25, 105). Additionally, IgA levels generally peak at 14-18 days after the first dose, increase slightly for one week after the second dose, but decrease thereafter (96, 102–105). In contrast to the significant increases in IgG levels after the second dose, studies have shown that IgA levels in milk do not rise further when measured > 18 days after the second dose (25, 101, 105).

**Table 2:** Summary of various studies evaluating vaccination during lactation and human milk antibodies with regards to the mRNA-1273 and BNT162b2 vaccines including sample size of lactating women, timepoints measured, and mean infant age.

Author	Vaccines	Measured antibodies (Ab) in human milk	Sample size (lactating women)	Timepoints	Mean infant age	Findings overall	Findings on milk IgG	Findings on milk IgA
Kelly et al. (102)	BNT162b2	Anti-spike IgG and IgA Ab levels	5	1. Pre-vaccine 2. 10-19 days post vaccination 3. 20-29 days post vaccination 4. 30-39 days post vaccination 5. >40 days post vaccination	9.8 months	-Both IgG and IgA levels were increased post vaccination	-Anti-spike IgG remained significantly increased 20 days post dose 1 to >40 days compared to pre-vaccine levels	-Anti-spike specific IgA were significantly increased 2 weeks post dose 1 to >40 days compared to pre-vaccine levels, although a decreasing level of mean IgA was observed at >40 days post dose 1
Perl et al. (103)	BNT162b2	Anti-spike IgG and IgA Ab levels	84	Pre-vaccine and weekly samples up to 6 weeks after first dose.	10.32 months	-Both IgG and IgA levels remained elevated in human milk 6 weeks post vaccination	-Mean anti-COVID specific IgG levels were low until week 3, and dramatically increased at week 4 and remained elevated at weeks 5 and 6	-Mean anti-COVID specific IgA levels increased significantly at 2 weeks post-first dose, decreased before the 2nd dose, and increased sharply 1 week post-second dose at week 4. -IgA levels remained elevated throughout the rest of the time points although steadily decreased.
Rosenberg-Friedman et al. (104)	BNT162b2	Anti-spike and RBD IgG and IgA Ab levels compared with a pre-pandemic control population	10 healthcare workers	1. 7 days post-first dose 2. 14 days post-first dose 3. 7 days post-second dose 4. 14 days post-second dose	5.13 months	-IgG: IgA ratios were calculated and suggested that IgA was the greatest at all time points, although the ratio increased significantly at 7 and 14 days post second dose, suggesting an increase in IgG over time post second dose. IgG and IgA levels increased at each time point and stopped increasing on 14 days post-second dose. - IgA production rate decreased 14 days post-second dose. IgG peaked at 14 days post-second dose whereas IgA showed a small decline at 14 days post-second dose.	-Anti-spike IgG at 7 days after first dose did not increase significantly compared to the controls, although increased significantly on day 14. Levels peaked on 7 days post second dose. -Anti-RBD IgG had a similar trend as above	-Anti-spike IgA increase significantly compared to controls 14 days after first dose. Levels peaked 7 days after second dose. -Anti-RBD IgA had a significant increase 7 days post second dose compared to controls.
Gray et al. (96)	mRNA-1273 and BNT162b2	Anti-spike and RBD IgG, IgA, and IgM Ab levels	31	1. Before first dose 2. After 1st dose: day of and before the 2nd dose 3. 2-6 weeks post-second dose	7.3 months (median)	-A significant increase of COVID specific IgG, IgA, and IgM was measured after first and after second dose compared to baseline.	-Increase in IgG was measured after second dose suggesting the boost facilitated an increase in transfer of IgG to human milk.	-IgA transfer in human milk did not increase after second dose compared to IgA levels after first dose.
Young et al. (105)	mRNA-1273 and BNT162b2	Anti-RBD IgG and IgA Ab levels	30	1. pre-vaccine 2. 18 days post-first dose 3. 18 days post-second dose 4. 90 days post-second dose	7.5 months	-Both IgG and IgA levels were increased post vaccination	-Large increase in IgG 18 days post-first dose and an additional increase 18 days after the second dose. It was followed by a decline at 90 days post-second dose.	-IgA levels increased at 18 days post-first dose, and didn’t further increase post-second dose
Golan et al. (25)	mRNA-1273 and BNT162b2	Anti-RBD IgG and IgA Ab levels in human milk and IgG and IgM in serum	50	1. Pre-vaccine 2. After first dose: day of and before the second dose 3. 4-10 weeks after the second dose	4.7 months (median)	-Both IgG and IgA levels were increased post vaccination -IgG levels were positively correlated between blood and milk between 4-10 weeks after the second dose	-IgG levels increased after the first dose and had a greater increase after the second dose.	- IgA levels significantly increase after the first dose, with no further increase 4-10 weeks after second dose.
Lechosa-Muñiz et al. (79)	BNT162b2, mRNA-1273 and ChAdOx1-S	Anti-RBD IgG and IgA Ab levels in human milk and serum	110	30 days after the second dose of the vaccine (or after first dose for ChAdOx1-S)	15.9 months	Significantly higher levels of IgG and IgA were found after mRNA-based vaccine vs. ChAdOx1-S.		
Selma-Royo et al. (80)	BNT162b2, mRNA-1273 and ChAdOx1	Anti-RBD IgG and IgA Ab levels in human milk	86	pre-vaccination, 1 week, 2 weeks, and 3–4 weeks post the 1st dose of vaccine; 1 week, 2 weeks, and 3–4 weeks post 2nd dose.	11-14.3 months	-Significant increase in IgA and IgG in milk with higher levels after second dose. -Antibody levels depend on vaccine type.	-IgG levels increased after the first dose with greater increase after the second dose.	- IgA levels after vaccination were lower compared to milk from COVID-19-infected women.

Studies on the association between blood and milk levels after SARS-CoV-2 vaccination during lactation have found a positive correlation between serum and human milk SARS-CoV-2 IgG levels measured at 4-10 weeks after second dose (25, 106). Interestingly, one study measured milk IgG and IgA in pregnant women who were vaccinated for both SARS-CoV-2 and TdaP during pregnancy and found similar levels between anti-Spike (SARS-CoV-2) antibodies and anti-tetanus toxoid (TT) antibodies (104). These findings further strengthen our knowledge about the mechanism and absolute level of transferred IgG antibodies from the serum to human milk *via* FcR transfer in the mammary gland (107).

Mothers who were infected with COVID-19 during pregnancy or lactation had a universally rapid anti-SARS-CoV-2 IgA secretion in human milk, lasting >90 days after diagnosis. In contrast, vaccination during pregnancy or lactation results in a robust anti-SARS-CoV-2 IgG secretion to milk with a less dominant IgA response (97, 105, 107–109). Though antibody functional responses may be similar after SARS-CoV-2 vaccination vs infection, as was demonstrated by comparable levels of neutralizing antibodies (97, 105). These findings suggest that exposure through natural infection leads to increased secretion of mucosal related IgA antibodies in mucosal organs, such as the mammary gland, which may be a distinct immune response than what is generated after mRNA-based vaccines. In animal models, additional intranasal vaccination induces mucosal boost immunity in addition to the systemic immunity that is induced after mRNA-based vaccines (110). Approaches boosting mucosal immunity may be useful to increase secretion of antibodies to human milk, however further research is needed in this area.

Similar to other vaccinations and infections, there is limited data on the presence SARS-CoV-2 antigen-specific human milk immune cells on infant protection against disease (111). Using animal models, it was shown that cells from milk can survive the digestive tract and can traffic into infant organs (112, 113). Interestingly two recent studies have demonstrated the presence of SARS-CoV-2 specific Spike-reactive T cells in human milk after vaccination (111, 114). However, it is unknown if human milk cells provide immune protection to the respiratory tract or gastrointestinal tract of human infants or if they are taken up in the infant gut into systemic circulation. The role of these antigen-specific immune cells in human milk in regard to infant protection requires further study.

### Protection of Infants

Further studies are needed to evaluate the protective effects of breastfeeding and milk SARS-CoV-2 antibodies against COVID-19 infection in infants. Exclusively breastfed infants usually consume human milk every 1-3 hours, providing them with frequent doses of milk antibodies. Upon weaning, milk antibodies decay rapidly in the infant, and this mode of passive immunity ends. Neonates and infants with COVID-19 often present with gastrointestinal symptoms (115). However, there is limited information to date on whether SARS-CoV-2 achieves gastrointestinal viral invasion or whether SARS-CoV-2 causes bystander mucosal inflammation that contributes to these symptoms (116–118). Interestingly, anti-SARS-CoV-2 IgA and IgG have been detected in one-third of 24 infant stool samples after maternal vaccination (119). Further studies are needed to determine the impact of local mucosal protection by human milk derived SARS-CoV-2 antibodies in the infant gut.

### COVID-19 Vaccines Safety During Lactation

COVID-19 vaccination for lactating women is recommended by the Centers for Disease Control and Prevention (CDC) to reduce the risk of complications from COVID-19, and the World Health Organization (WHO) recommends continuing of breastfeeding after vaccination (120, 121). Maternal vaccination during lactation protects the mother from severe COVID-19 disease and as discussed above may also protect the infant. A large survey-based study including over 10,000 lactating individuals found minimal disruption of lactation after vaccination (around 2% of the individuals), with 6% of individuals reporting decrease in milk supply (122). Reduction in milk supply was reported in 5-7% of the women, which was more common after the second dose. Most symptoms resolved within 24-72 hours after vaccination (25, 122, 123). Symptoms in the breastfed infant in the short term after maternal vaccination were reported in 2-7% the cases, with sleepiness and fussiness being the most common symptom (25, 122, 123). Other symptoms such as fever and gastrointestinal symptoms were reported in 1-2% of the infants (25, 122, 123). Few studies examined transfer of vaccine particles to human milk after vaccination (101, 124) and found minimal transfer of vaccine mRNA to human milk in less than 2% of the samples (out of 309 samples examined). In addition, a single study measured polyethylene glycol (PEG) which is present in the lipid nanoparticles of the mRNA-based vaccines in milk and found no significant increase in PEG in milk after vaccination (25). It is not clear whether the infant symptoms reported are specifically related to vaccine particle transfer, and further research is needed in this area. There is a lack of clinical trials that carefully examine infant side effects after vaccination in this vulnerable population of lactating dyads. Future trials should include these populations and outcomes. However, based on the data collected so far in multiple prospective studies, the benefits of vaccination outweigh the risk for mother and her infant.

### COVID-19, Lactation, and Equity Issues

Despite the known maternal and infant health benefits of breastfeeding and vaccination, significant inequities persist among the most vulnerable groups that are presented with unique challenges to lactation support and vaccine access. There are a lack of studies examining barriers to breastfeeding during the COVID-19 pandemic. Access to commercial tele-lactation companies offering online lactation support is limited especially for those who have lost their jobs and may not be able to afford lactation or internet services (117). It is essential to provide resources to the communities and populations purposively marginalized. For instance, in certain parts of large cities with previous inequitable health care access, such as the South Side of Chicago, the COVID-19 pandemic has exacerbated the reduction of open hospitals (118). Hospitals are typically the primary source of breastfeeding education and in communities with already low rates of breastfeeding. Barriers of marginalized populations are being aggravated rather than reduced during the pandemic. There is also inadequate funding to lactation services in institutions and agencies. Addressing systemic and structural barriers and increasing funding to lower resourced communities can begin to reduce health care disparities by providing essential services, such as open hospitals and consistent breastfeeding education so that families understand the short- and long-term importance of vaccination and breastfeeding.

## Discussion

### Future Directions

The studies presented here have demonstrated the benefits of influenza, pertussis, and SARS-CoV-2 vaccination for pregnant and lactating individuals and the presence of anti-pathogens antibodies in human milk following vaccination. Further epidemiological studies are needed to determine the level of disease protection to infants against COVID-19 provided by maternal vaccination through human milk. Additionally, the quantity of human milk required to be ingested to confer a protective effect in an infant is unknown. To address this question, detailed study of infant feeding patterns is needed to distinguish between various quantities and patterns of human milk consumption. Current studies usually compare only exclusively breastfed to nonexclusively breastfed infants as a group. In addition, studies that measure the durability of milk antibodies in infant mucosal surfaces, such as the oropharynx, are necessary to better understand the protection of milk antibodies against pathogens that are transmitted *via* these organs.

Longitudinal studies to evaluate the persistence of human milk antibodies after vaccination, and the effect of a third and fourth mRNA-based vaccine doses on human milk are needed. Similarly, long-term follow up on infants of COVID-19 vaccinated mothers is needed as the pandemic evolves to provide more data on protection of these infants with continued breastfeeding.

In summary, while the immunologic benefits of breastfeeding have long been promoted, there is still much to learn regarding the dynamics of immune responses during lactation. More work is needed to understand the precise mechanisms of immune protection seen in breastfed infants. However, the potential benefits of breastfeeding and human milk are nullified if there is not equitable access and support for lactation, particularly in vulnerable communities. Research and financial support for qualitative studies and community-engaged programs are needed to improve advocacy for education and resources in lactating communities of color.

## Author Contributions

SH and YG wrote the first draft. All authors revised the manuscript. All authors contributed to the article and approved the submitted version.

## Funding

SLG- K08 AI141728/AI/NIAID NIH HHS/United States; IVA- K12 HD052163/HD/NICHD NIH HHS/United States; MP- K23 AI127886/AI/NIAID NIH HHS/United States; YG-Weizmann Institute of Science -National Postdoctoral Award Program for Advancing Women in Science and Human Frontier Science Program.

## Conflict of Interest

The authors declare that the research was conducted in the absence of any commercial or financial relationships that could be construed as a potential conflict of interest.

## Publisher’s Note

All claims expressed in this article are solely those of the authors and do not necessarily represent those of their affiliated organizations, or those of the publisher, the editors and the reviewers. Any product that may be evaluated in this article, or claim that may be made by its manufacturer, is not guaranteed or endorsed by the publisher.
